# Achieving Stable Nitritation for Mainstream Deammonification by Combining Free Nitrous Acid-Based Sludge Treatment and Oxygen Limitation

**DOI:** 10.1038/srep25547

**Published:** 2016-05-06

**Authors:** Dongbo Wang, Qilin Wang, Andrew Laloo, Yifeng Xu, Philip L. Bond, Zhiguo Yuan

**Affiliations:** 1 Advanced Water Management Centre (AWMC), The University of Queensland, QLD 4072, Australia

## Abstract

Stable nitritation is a critical bottleneck for achieving autotrophic nitrogen removal using the energy-saving mainstream deammonification process. Herein we report a new strategy to wash out both the *Nitrospira* sp. and *Nitrobacter* sp. from the treatment of domestic-strength wastewater. The strategy combines sludge treatment using free nitrous acid (FNA) with dissolved oxygen (DO) control in the nitritation reactor. Initially, the nitrifying reactor achieved full conversion of NH_4_^+^ to NO_3_^−^. Then, nitrite accumulation at ~60% was achieved in the reactor when 1/4 of the sludge was treated daily with FNA at 1.82 mg N/L in a side-stream unit for 24 h. Fluorescence *in-situ* hybridization (FISH) revealed FNA treatment substantially reduced the abundance of nitrite oxidizing bacteria (NOB) (from 23.0 ± 4.3 to 5.3 ± 1.9%), especially that of *Nitrospira* sp. (from 15.7 ± 3.9 to 0.4 ± 0.1%). Nitrite accumulation increased to ~80% when the DO concentration in the mainstream reactor was reduced from 2.5–3.0 to 0.3–0.8 mg/L. FISH revealed the DO limitation further reduced the abundance of NOB (to 2.1 ± 1.0%), especially that of *Nitrobacter* sp. (from 4.9 ± 1.2 to 1.8 ± 0.8%). The strategy developed removes a major barrier for deammonification in low-strength domestic wastewater.

It is increasingly recognized that wastewater treatment plants (WWTPs) should be transformed to resource recovery facilities^1^. Indeed, there is currently a strong emphasis on the development of energy-efficient and energy-producing technologies for wastewater treatment^2^,^3^. Autotrophic nitrogen removal (i.e., deammonification), which consists of partial nitritation and anaerobic ammonium oxidation (Anammox), requires much less energy than the conventional nitrification-denitrification process. It also enables the upfront separation of organic carbon to enhance bio-energy recovery^4^,^5^,^6^. For example, one process configuration with two stages (i.e., the A/B process) based on deammonification has been proposed for mainstream wastewater treatment^5^,^7^. Wastewater is first fed to the A-stage, where most of the organic matter is captured by biomass/sludge, which is subsequently converted to bioenergy through anaerobic digestion^7^,^8^. The effluent of the A-stage, with relatively low chemical oxygen demand (COD) but high nitrogen, is further treated by mainstream deammonification at the B-stage^5^. This process configuration concurrently achieves maximum energy recovery and desirable nutrient removal^4^,^5^,^8^, with the deammonification process playing a central role^9^,^10^,^11^.

The autotrophic nitrogen removal can be achieved in either one-stage or two-stage processes^6^,^9^,^11^,^12^. Compared with the one-stage process, the two-stage process can enhance the activity of anammox due to the absence of oxygen, though it needs an additional nitritation reactor for the bioconversion of ammonium to nitrite^12^,^13^. To date, both processes have been implemented for autotrophic nitrogen removal from anaerobic sludge digestion liquors at full-scale plants^6^, but mainstream deammonification is still at its infancy^5^,^14^. The major barriers hindering the application of deammonification to mainstream wastewater treatment are: 1) low growth rates of Anammox organisms, 2) competition between denitrifiers and anammox organisms, and 3) unstable nitritation^5^,^15^. Of these barriers, selective retention of ammonia-oxidizing bacteria (AOB) over nitrite-oxidizing bacteria (NOB) to achieve stable nitritation is considered the biggest challenge due to the similar growth kinetics of AOB and NOB^5^,^9^,^10^.

Several parameters, such as pH, temperature, dissolved oxygen (DO), sludge retention time (SRT), free ammonia and free nitrous acid (FNA), have been reported to affect the AOB-NOB growth kinetics^5^,^16^,^17^,^18^. A combination of these factors have led to the relatively easy establishment of stable nitritation in sidestream reactors treating anaerobic sludge digestion liquor^16^,^19^. However, it remains a challenge to establish stable nitritation in mainstream wastewater treatment, even though extensive efforts have been made in search of effective solutions^5^. This is because, in comparison to anaerobic sludge digestion liquor, among other differences mainstream wastewater has much lower nitrogen concentration and lower temperature. These differences make it much more difficult to exert selective pressures against NOB, while still allowing AOB to grow.

Blackburne *et al.* presented a strategy for eliminating NOB in a nitrifying reactor^20^. This was achieved by combining low DO (0.4 mg/L) with a short SRT of 2.4 days, resulting in ~90% nitrite accumulation. However, it is difficult to implement this strategy in practice, as a SRT of 2.4 days results in a system with biomass levels and reaction rates that are too slow for the application. It was shown that NOB was eliminated because of its low affinity with oxygen in comparison with AOB^20^. However, Regmi and co-workers provided contradicting evidence showing that a high DO concentration (>1.5 mg/L) could also provide competitive advantage to AOB over NOB^14^.

Recently, Wang *et al.* proposed a novel approach to exert selection pressure against NOB in a nitrification-denitrification system through sludge treatment in the sludge returning line^8^. The strategy is based on recent findings that FNA, the protonated form of nitrite, has strong biocidal effects on a broad range of microorganisms at parts per million (mg N/L) levels^21^,^22^, and more importantly, it has a stronger biocidal effect on NOB than on AOB^8^. Although FNA levels in a mainstream bioreactor are usually negligible, it can be produced at parts per million levels from anaerobic sludge digestion liquor, which contains ammonium at around 1 g NH_4_^+^-N/L^16^. Wang *et al.* incorporated an FNA-treatment unit in the sludge recycling line to treat 22% of the sludge from the bioreactor daily at an FNA concentration of 1.35 mg/L for 24 h. Within two weeks they rapidly established nitrogen removal via the nitrite pathway in a nitrification-denitrification bioreactor treating domestic wastewater, with a nitrite accumulation ratio (NO_2_^−^-N/(NO_2_^−^-N + NO_3_^−^-N) of 81.5 ± 0.1%^8^.

A synergetic effect between FNA treatment and the competition between NOB and denitrifiers in the bioreactor was hypothesized to cause selection against the NOB^8^. In the nitrification-denitrification bioreactor, the ability of NOB to compete with denitrifiers for nitrite would be weakened following regular FNA treatment due to its reduced abundance and activity. Such a competition with denitrifiers does not exist to the same extent in a mainstream nitritation reactor proceeding the Anammox reactor due to the low influent COD concentration in the A-stage effluent. This means that it will be much more challenging to achieve stable nitritation in a reactor without or with low levels of denitrification. In the absence of competitors, kinetic selection would play a much more important role in the elimination of NOB in this case.

*Nitrospira* sp. and *Nitrobacter* sp. are widely regarded as the two major types of NOB present in WWTPs. In previous investigations, they are often considered as one functional group^23^,^24^,^25^. However, *Nitrospira* sp. and *Nitrobacter* sp. exhibit different growth characteristics. For example, *Nitrobacter* sp. are considered to be r-strategists with low affinity for oxygen, while *Nitrospira* sp. are K-strategists with higher oxygen affinity^14^. This may explain the different results reported to date regarding the effects of DO on NOB elimination and indicates that different strategies may be required to suppress the two groups. Thus, stable nitritation may be achieved in mainstream wastewater treatment if different strategies that can effectively wash out either *Nitrospira* sp. or *Nitrobacter* sp. are combined. Nevertheless, such a combined strategy developed for mainstream processes has seldom been reported before.

The aim of this study was to develop an effective strategy to establish stable nitritation in a mainstream nitritation reactor, through FNA-based sludge treatment with possible integration with DO control. A nitrifying reactor receiving synthetic wastewater containing ammonium at 57 mg N/L but no COD was used in the study. The influence of sludge treatment with FNA on nitrite accumulation and on the suppression of NOB was first investigated. A low DO control strategy was then implemented to further enhance nitrite accumulation. Finally, aeration length control, to stop aeration when 50% of the influent ammonium was converted, was implemented to produce an effluent suitable for the Anammox reaction^26^. The strategy presented here removes the biggest barrier for autotrophic nitrogen removal from mainstream wastewater.

## Results

### FNA concentration, FNA treatment time, and sludge treatment ratio selected for the FNA treatment unit

The mainstream reactor was operated in steady state for more than one year with almost 100% conversion of ammonium to nitrate before the batch tests commenced. The AOB and NOB populations measured with the specific FISH probes accounted for 58 ± 5% and 23 ± 4% of total bacteria, respectively (Figure S1, Supporting Information).

In batch test set I, both AOB and NOB activities decreased with the increase of FNA level, and the NOB activity decrease was much greater than the AOB activity decrease at all FNA treatment levels investigated (Table 1). With an increase of the FNA treatment from 1.34 to 1.82 mg N/L, the relative activity (expressed as % of the original) of NOB decreased from 75.3% to 43.5%. When 3.64 mg N/L of FNA was applied, the NOB activity decreased to 6.7% of the original. In these batch tests the measured particle size distribution (d_50_) decreased from 240 μm (before FNA treatment) to 138 μm and 23 μm after FNA treatment at 1.82 and 3.64 mg N/L, respectively (Figure S2, Supporting Information). This indicated that an FNA level of 3.64 mg N/L may deteriorate the settling properties of the sludge and cause significant loss of biomass during the long-term operation of the mainstream reactor. Thus, both 3.64 and 1.82 mg N/L were selected for the subsequent batch tests for further optimization, mainly for their effectiviness of reducing NOB activity to a very low level.

Batch test set II showed that the treatment time of FNA affected both AOB and NOB activities (Table S1, Supporting Information). On the basis of the measured AOB and NOB activities, 24 h was selected as the treatment time for the long-term operation of the FNA treatment unit in Phases II-VI, while 25% of sludge in the mainstream reactor was decided to be treated daily, giving an average ‘recovery time’ of 4 days after the treated sludge is returned to the reactor.

Batch test set III further verified that although nitrite and pH were different among these FNA treated reactors, FNA was the main contributor to the decreased NOB activity. This is because the NOB activity in both the pH 6 and the nitrite only treatment reactors did not vary significantly after 24 h treatment (p > 0.05), as compared with the original activity (Table S2, Supporting Information).

### Mainstream reactor performance in all phases

**Figure 1:**
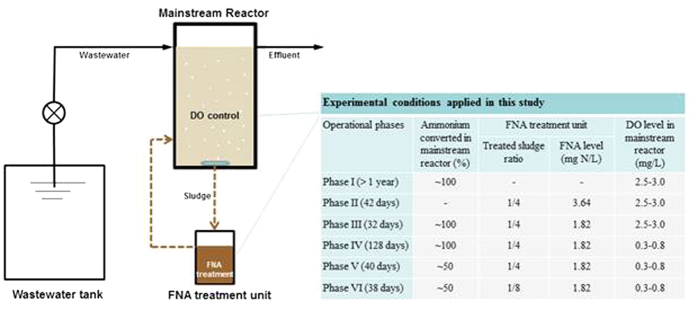
Schematic diagram of the experimental system and the operational conditions applied in six phases of the study.

**Figure 2:**
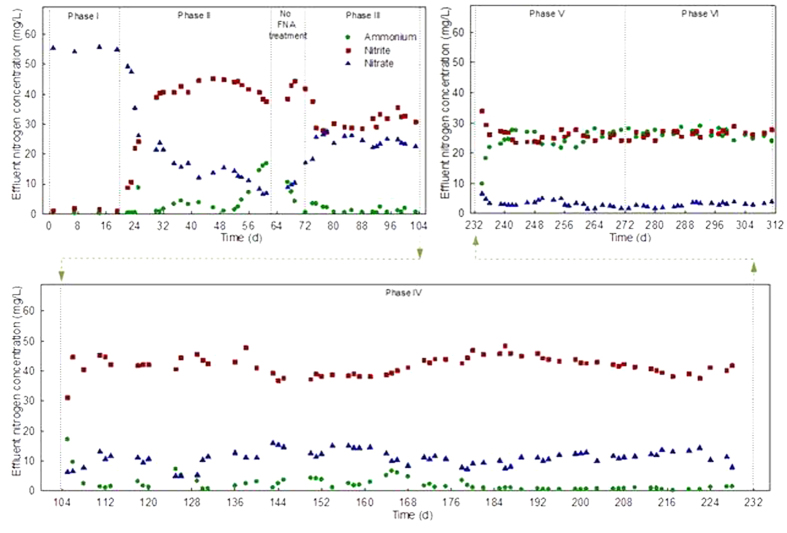
The long-term variations of effluent ammonium, nitrite, and nitrate in the mainstream reactor under the different operational conditions.

To establish stable nitritation in the mainstream reactor, different conditions were tested in different phases of the study, as shown in Fig. 1. Figure 2 shows the long-term variations of the effluent ammonium, nitrite, and nitrate concentrations in the mainstream reactor under different operational conditions, with the steady-state data in all phases except for Phase II (no steady state achieved) further summarized in Table 2. The TSS and VSS concentrations in both the mainstream reactor and the effluent were relatively stable in all phases except for Phase II (Figure S3, Supporting Information).

#### Phase I: Initial Operation of the Fully-Nitrifying Mainstream Reactor

In this phase, ammonium was almost fully converted to nitrate at the end of the aerobic period. (Fig. 2, Table 2, and see Figure S4A, Supporting Information, for typical cycle data).

#### Phase II: Influence of Sludge Treatment with FNA at 3.64 mg N/L on Reactor Performance

As batch tests showed that FNA at 3.64 mg N/L almost fully inactivated NOB (Table S1, Supporting Information), this level of FNA was first tested. Upon implementation of the treatment, effluent nitrate decreased sharply accompanied by a dramatic increase of effluent nitrite concentration (Fig. 2). The highest effluent nitrite concentration was 45.1 mg/L, representing 74% of the influent ammonium concentration. However, after about one-month operation, the effluent ammonium concentration increased gradually, with 16.8 mg/L ammonium measured at the end of this phase on day 61 (Fig. 2 and Figure S4B, Supporting Information). Moreover, the VSS concentration in the mainstream reactor decreased from 390 mg/L (before FNA treatment, Phase I) to 45 mg/L after 30 d treatment, whereas the effluent VSS level increased from 14 mg/L (Phase I) to 18–30 mg/L (Phase II) (Figure S3, Supporting Information). Although FNA treatment at 3.64 mg N/L could effectively achieve nitrite accumulation, it caused substantial loss of biomass via the effluent. This was likely because of the disruption of sludge flocs by the FNA treatment (Figure S2, Supporting Information). Thus, Phase 2 was terminated.

#### Phase III: Influence of Sludge Treatment with FNA at 1.82 mg N/L on Reactor Performance

Following Phase II the FNA treatment was terminated for a 10 d recovery period, then FNA treatment at 1.82 mg N/L was implemented. This level of FNA treatment did not result in deterioration of ammonium conversion, with the effluent ammonium concentration remaining below 1 mg/L after about one-week adaption (Fig. 2). In addition, the effluent VSS lowered to around 14 mg/L (Figure S3, Supporting Information), comparable to that in Phase I, and the VSS in the mainstream reactor increased from ~45 mg/L in Phase II to ~170 mg/L in this phase. However, in comparison to Phase II, the effluent nitrite concentration decreased to 34.9 ± 0.5 mg/L (58.5 ± 0.3% of the effluent total nitrogen) whereas the effluent nitrate level increased to 23.9 ± 0.8 mg/L (40.2 ± 0.7% of effluent total nitrogen) (Table 2).

#### Phase IV: Influence of FNA Treatment in Combination with DO Limitation on Reactor Performance

Although substantial nitrite accumulation was achieved in Phase III, 23.9 ± 0.9 mg/L of nitrate was present in the effluent from the mainstream reactor, which would not be removed by a subsequent Anammox process. To reduce the effluent nitrate concentration, the oxygen concentration in the mainstream reactor was decreased from 2.5–3 mg/L, the previous phase level, to 0.3–0.8 mg/L, with the hypothesis here that a lower DO would exert additional selection pressure against NOB^5^,^20^. The effluent nitrate decreased from 40.2 ± 0.7% in Phase III to 19.5 ± 1.1% (Fig. 2, Table 2) of the total nitrogen concentration, while the effluent nitrite level increased from 58.5 ± 0.3% to 78.9 ± 1.1%. In comparison, the effluent ammonium concentration remained at a low level of 1.6 ± 0.3%. The application of a low DO level significantly enhanced nitrite accumulation. The possible mechanism is further discussed below.

#### Phase V: Aeration Length Control to Achieve 50% of Influent Ammonium Conversion

It is well-known that the ideal molar ratio of nitrite to ammonium is roughly at 1:1 for the Anammox process^27^. Consequently, we aimed to achieve a molar ratio of 1:1 between nitrite and ammonium in the effluent of the mainstream reactor. In this phase, aeration was switched off when an estimated (based on oxygen consumption) 50% ammonium conversion was achieved. A molar ratio of nitrite to ammonium of approximately 1:1 (47.6 ± 0.5% nitrite vs. 48.6 ± 1.5% ammonium) was indeed achieved (Fig. 2, Table 2). In addition, the effluent nitrate level was further reduced to 3.8 ± 1.0% (2.07 ± 0.55 mg/L) from the previous level of 19.5 ± 1.1% (10.8 ± 0.6 mg/L).

#### Phase VI: Combined FNA Treatment and Mainstream DO Limitation with Reduced Sludge Treatment Frequency

Reducing the sludge treatment frequency could reduce the operational costs of a full-scale system based on the mainstream reactor operation (as is further discussed below). The sludge treatment frequency applied in Phases II–V was based on the batch test results. The biomass composition applied in the batch tests was different to that detected in Phase V (Table 3), and there is the possibility to reduce the treatment frequency without losing the mainstream reactor performance.

When the ratio of treated sludge was reduced from 1/4 (Phase V) to 1/8 (Phase VI), the reactor effluent ammonium, nitrite, and nitrate levels were not affected significantly (Fig. 2, Table 2). This indicated that it is feasible to achieve partial nitritation with only 1/8 of the sludge in the mainstream reactor treated on a daily basis. It should be emphasized that there is no evidence showing that this sludge treatment frequency is optimal, and indeed it may be further reduced in future experiments.

### How did this combined strategy achieve stable nitritation in the mainstream reactor?

The populations and activities of AOB and NOB in the mainstream reactor were measured in all phases. Table 3 summarizes the changes of AOB and NOB abundances in the mainstream reactor, with example FISH images presented in Figure S1 (Supporting Information).

The population of NOB in the mainstream reactor accounted for only 5.3 ± 1.9% of the total bacteria at steady-state in Phase III, which was much less than that in Phase I (23.0 ± 4.3% of total bacteria). Correspondingly, the NOB activity largely decreased sharply from 49.4 ± 3.1 (Phase I) to 24.9 ± 1.9 mg N/g VSS·h (Phase III). The abundance and activity data both clearly show that the FNA treatment resulted in strong suppression of NOB. On the contrary, the AOB abundance increased from 58.5 ± 5.4% (Phase I) to 71.7 ± 7.2% (Phase III) of the bacteria. This increase detected could be due to the decrease of the relative NOB abundance rather than an absolute increase in AOB numbers. The AOB activities did decrease slightly from 67.2 ± 3.4 (Phase I) to 59.5 ± 3.8 mg N/g VSS·h (Phase III). It was evident that FNA treatment at 1.82 mg N/L had relatively minor suppression effect on the AOB activity. When a DO limitation was imposed in combination with FNA treatment (Phase IV), the NOB abundance and activity further decreased to 2.1 ± 1.0% and 9.8 ± 2.6 mg N/g VSS·h, respectively. This clearly indicates that low DO exerted an additional selection pressure against *Nitrobacter* (the dominating NOB following FNA treatment), due to its relatively low oxygen affinity. When the reactor was achieving about 50% of ammonium conversion, the abundance of NOB decreased to below 1% in both Phase V (1/4 sludge treated) and Phase VI (1/8 sludge treated). The decreases in NOB populations were accompanied by increases in the AOB populations, but relatively constant AOB activity. These suggest that these FNA treatments and the DO limitation did not have pronounced effects on the AOB growth or activity. It should be noted that the populations of *Nitrospira* and *Nitrobacter* were less than 1% of the total bacteria in Phases V and VI whereas the corresponding NOB activity was around 15% of the original, suggesting that apart from *Nitrospira* and *Nitrobacter* other NOB also existed in the nitritation reactor.

Two types of NOB, namely *Nitrospira* sp. and *Nitrobacter* sp., have been found to be the main NOB in WWTPs^28^. To differentiate the potential different influences of FNA treatment and DO limitation on these two types of NOB, we further investigated the changes of *Nitrospira* sp. and *Nitrobacter* sp. abundance in the different phases. Representative FISH micrographs are detailed in Figure S5, in the Supporting Information. In Phase I, *Nitrospira* sp. and *Nitrobacter* sp. represented 15.7 ± 3.9% and 7.3 ± 2.6%, respectively, of the total bacteria. The relative abundance of these two groups is in general agreement with that found in full-scale WWTPs^28^,^29^,^30^.

In Phase III, the abundance of *Nitrobacter* sp. dropped by 1/3 from 7.3 ± 2.6% to 4.9 ± 1.2% of the total bacteria respectively (Table 3). In comparison, *Nitrospira* sp. was almost completely eliminated (from 15.7 ± 3.9% to 0.4 ± 0.1%) in this phase and remained at very low or non-detectable levels in all remaining phases (Table 3). These results indicate that FNA is an effective method to wash out *Nitrospira* sp., which is typically a dominant NOB group present in nitrifying WWTPs. While it appears that *Nitrobacter* sp. are more tolerant to FNA than NOB, biological details of their greater tolerance is presently unknown. It has previously been detected that *Nitrobacter* sp. are adapted to higher nitrite concentrations whereas *Nitrospira* sp. are favored in lower nitrite levels^28^.

Lowering the reactor DO from 2.5–3.0 mg/L to 0.3–0.8 mg/L in Phase IV resulted in a further significant decrease in the abundance of *Nitrobacter* sp. from 4.9 ± 1.2% to 1.8 ± 0.8%. This clearly suggests that DO limitation in the mainstream reactor can suppress the growth of *Nitrobacter* sp. When the 50% ammonium conversion strategy was implemented, *Nitrobacter* sp. further dropped to below 1% (while *Nitrospira* sp. remained non-detectable). This re-emphasizes that DO limitation and aeration length control could be an effective strategy to suppress *Nitrobacter* sp.

In summary, it can be concluded that stable nitritation in the mainstream reactor was achieved through the combined effects of FNA treatment and DO limitation (low DO and aeration length control). FNA was primarily responsible for the elimination of *Nitrospira* sp. while DO limitation resulted in the washout of *Nitrobacter* sp.

Our findings are consistent with literature reports that *Nitrobacter* sp. are r-strategists with a low oxygen affinity while *Nitrospira* sp. are K-strategists with a higher oxygen affinity^14^,^30^. That is, a high DO could provide competitive advantage for AOB over *Nitrospira* sp. while low DO levels are beneficial for washing out *Nitrobacter* sp. This could explain the inconsistent observations regarding the DO influence on NOB suppression previously reported in literature. For example, the NOB enriched in Regmi *et al.*, dominated by *Nitrospira* sp., was found to be more easily suppressed by the use of high DO^14^, while the NOB enriched in Blackburne *et al.*, dominated by *Nitrobacter* sp., was successfully suppressed with the use of low DO^20^. As such, DO control alone is unlikely an effective strategy in practical applications, as it can not simultaneously eliminate both *Nitrospira* sp. and *Nitrobacter* sp., both of which are present in WWTPs.

### What happens in the FNA treatment unit?

To reveal what happens in the FNA treatment unit, variations of total COD, soluble COD, nitrogen compounds, and activities of AOB and NOB were measured in the unit before and after FNA treatment at steady-state in Phase III (Table 4). No significant variation was detected in the nitrite and total COD concentrations before and after the treatment (p > 0.05). Ammonium and nitrate were always detected at very low levels. These results indicated that the bio-reactions of both nitrification and denitrification did not occur in the FNA treatment unit. Soluble COD concentration and the activities of AOB and NOB, however, were affected by the FNA treatment. It was found that the activities of AOB and NOB decreased by 21% and 53%, respectively. These results are in general agreement with the batch test results (Table 1). After 24 h treatment, the soluble COD concentration increased from 6 ± 2 mg/L to 327 ± 21 mg/L. The increase could be due to cell lysis and solublization of extracellular materials^31^,^32^. Nitrification relevant enzymes such as ammonia monooxygenase and nitrite oxidoreductase are cell membrane-bound enzymes and it is possible the homogeneity of the enzymes environment may be assisted by the adjacent extracellular polymeric substances. It is reported that extracellular polymeric substances are disrupted by FNA^31^,^32^, and that FNA may react directly with enzymes involved in metabolic processes^21^. Consequently, FNA could be acting here to both expose and directly inhibit these membrane-bound nitrification enzymes. To date, however, it is unknown why the same level of FNA causes different effects on the activities of AOB and NOB. These are interesting speculations and questions that require further investigation.

## Discussion

Presently, about 100 full-scale sidestream installations using nitritation/anammox are successfully operated worldwide. There is strong interest to more broadly apply the energy efficient technology to mainstream wastewater treatment^6^,^10^. Although numerous efforts have been made to remove the biggest barrier (i.e., stable nitritation) for mainstream treatment, it is still an un-resolved challenge. Isanta *et al.*^33^ reported that stable partial nitritation with effluent nitrate levels around 2.5 mg/L was achieved in an aerobic granular reactor for low-strength wastewater. However, it should be emphasized that the inoculum used did not contain the major type of NOB, *Nitrospira*. It remains largely unknown that the method reported in their work can effectively wash out both *Nitrospira* and *Nitrobacter*. This is to the best of our knowledge the first study successfully washing out both *Nitrospira* and *Nitrobacter* from low-strength wastewater treatment, which thereby addresses this biggest bottleneck for mainstream deammonification using a practicable engineering approach. The method was experimentally verified through both chemical and microbial analyses of both batch and long-term tests. Under the condition of 50% ammonium conversion (Phase V and Phase VI), an effluent nitrite to ammonium ratio of 1:1 could be stably achieved, with the effluent nitrate level being 5% (or less) of the total effluent nitrogen.

By combining low DO and low SRT controls, Blackburne *et al.*^20^ obtained about 90% of nitrite accumulation (NO_2_^−^-N/NO_x_^−^-N) in a nitrifying reactor treating low strength ammonium wastewater. However, the low SRT-based method used by Blackburne *et al.* inevitably requires high HRT to fulfil ammonium conversion, which largely diminishes the value of mainstream deammonification. The new FNA-based technology developed here does not have this drawback. The SRT was estimated to be approximately 8 d in Phase V and Phase VI, thus allowing adequate accumulation of biomass. Due to hydraulic limitations associated with our laboratory SBR, an HRT of 13.2 h was applied in this study, resulting in a low reactor VSS concentration of 100–110 mg/L. In practice, the VSS concentration can be substantially enhanced and the HRT decreased if a more efficient separation system, for example, a membrane bioreactor, is used for biomass retention. This remains to be investigated in future studies.

**Figure 3:**
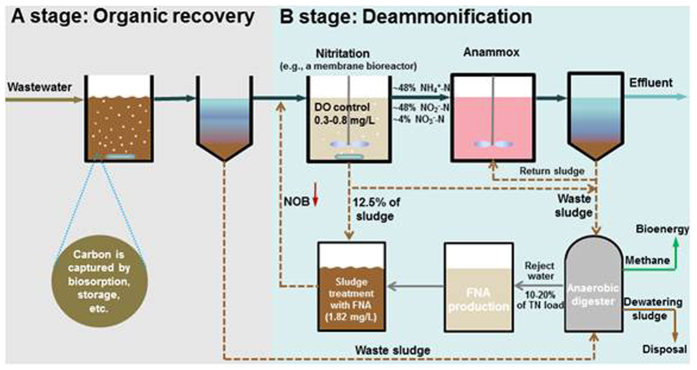
The conceptual operation of a WWTP with FNA-supported mainstream deammonification for enabling maximal energy recovery and desirable nutrient removal.

FNA is a renewable chemical that can be produced *in-situ* at WWTPs as a byproduct of wastewater treatment by nitritation of the anaerobic digestion liquor^16^,^22^. Generally, the ammonium in the anaerobic digestion liquor requires to be removed or converted, and nitrite is necessarily an intermediate^16^. Based on the results of this work, we propose to use this intermediate product to favorably manipulate the microbial community and activity in the mainstream reactor before its final removal as N_2_ (Fig. 3). This new FNA-based strategy creates a closed-loop wastewater management system for enabling maximum energy recovery and desirable nutrient removal simultaneously, thereby providing strong support for the on-going paradigm shift in wastewater management (i.e., from pollutant removal to energy recovery).

Here we present a closed-loop concept for the operation of a WWTP with the FNA-based method developed in this work (Fig. 3). Most organic carbon is first removed in the A-stage treatment through the mechanisms of bio-sorption and storage, and then the effluent of the A-stage, with a relatively low COD/nitrogen ratio is further treated at the B-stage by mainstream deammonification. To date, chemically enhanced primary treatment and high rate activated sludge have been explored for carbon capture in the A-stage^5^. It is reported that >80% of organic carbon and phosphorus could be removed in a high-rate reactor with SRT of 2–3 days and HRT of 0.5–1 day^7^. The captured organic carbon and waste activated sludge from B-stage are channeled to the anaerobic digester for methane production. The energy captured there, as methane, can be collected and further utilized, while the anaerobic digestion liquor is used for FNA production. Typically this anaerobic digestion liquor contains 0.8–1.5 g/L of ammonium, and more than 90% of this ammonium can be bio-converted to nitrite in a sidestream nitritation reactor^16^,^22^. To establish mainstream nitritation, this study demonstrated that no more 12.5% of biomass in the mainstream reactor needs to be treated daily with 1.82 mg N/L FNA in the sludge treatment unit for 24 h. The effluent of the mainstream nitritation reactor is then further treated in the subsequent anammox reactor before its final discharge. In such a WWTP design, with FNA-supported mainstream deammonification, maximal energy is recovered and desirable nutrient removal is achieved.

To this end, a desktop scaling-up study of a full-scale WWTP with a 200 000 population equivalent was carried out to economically evaluate this operational concept (Table S3, Supporting information). The methane yield in such a WWTP is estimated to be 4.7 times of that in the conventional WWTP with nitrification and denitrification (8 × 10^5^ vs 1.7 × 10^5^ kg CH_4_/y). Moreover, the required FNA concentration (i.e., 1.82 mg N/L) can easily be achieved through nitritation of the anaerobic sludge digestion liquor. Therefore, the proposed closed-loop operational concept for WWTPs is economically attractive and practically feasible. However, the values listed here should be considered indicative only and these need to be re-verified when applied to real WWTPs due to the different levels of solids, COD, and alkalinity in the A-stage effluent. It is known that real wastewaters are more complex than the synthetic medium prepared in this study. The effluent of A-stage should contain certain levels of organic carbon and solids, which may stimulate the growth of heterotrophic bacteria and affect the viscosity of sludge. As a result, the performance of the subsequent deammonification process and the effectiveness of the FNA treatment unit may be affected. In addition, the alkalinity of real wastewaters also varies. This variation is expected to affect the amount of acid required for FNA treatment. Therefore, the economic and technical analysis should be refined when full-scale data become available.

## Methods

### Reactor operation and overall experiment design

One lab-scale sequencing batch reactor with a working volume of 11 L was operated as the mainstream reactor in the laboratory at room temperature (22 ± 1 °C). This reactor received wastewater containing ammonium at 57 mg N/L, a concentration within the typical range of nitrogen in domestic wastewater. No organic carbon source was supplied in the synthetic wastewater in order to eliminate potential competition from denitrifiers. To establish stable nitritation in the mainstream reactor, its operation was divided into six phases, as shown in Fig. 1.

### Wastewater composition

Synthetic wastewater was used in this study and prepared every two days. The synthetic wastewater contained domestic level of ammonium without organic carbon source, with a composition of (per liter): 0.2949 g of NH_4_HCO_3_ (57 mg NH_4_^+^-N), 0.33 g NaHCO_3_, 0.184 g of NaCl, 0.072 g of NaH_2_PO_4_·H_2_O, 0.035 g MgSO_4_·7H_2_O, 0.029 g KCl and 0.3 mL of a trace element stock solution prepared as described previously^34^.

### Initial operation of the fully-nitrifying mainstream reactor (Phase I)

The reactor was seeded with sludge taken from a domestic wastewater treatment plant in Brisbane, Australia. The reactor was operated with four cycles daily. Each cycle started with a 90 min aerobic feeding period, during which 5 L of synthetic wastewater was pumped into the reactor, followed by 210 min aerobic reaction, 50 min settling and 10 min decanting periods. DO was controlled between 2.5 and 3.0 mg/L in the feeding and aerobic periods with a programmed logic controller, while the pH in these two periods was controlled at 7.5 by dosing 1 M NaHCO_3_. The reactor was constantly mixed with a magnetic stirrer except for the settling and decanting periods. In the decanting period, 5 L of the supernatant was discharged from the reactor, resulting in a hydraulic retention time (HRT) of 13.2 h. No sludge wasting was carried out. The SRT was estimated to be approximately 12 d during steady-state operation based on the measured total suspended solids (TSS) and effluent TSS concentrations. In this phase, sludge treatment with FNA was not implemented.

### Batch experiments

To determine suitable conditions of FNA concentration, treatment time, and treatment frequency applied to the FNA treatment unit, the following two sets of batch tests were performed at room temperature of 22 ± 1 °C.

Batch test set I tested the effect of different levels of FNA treatment on AOB and NOB activities to select potentially suitable FNA levels. Eight FNA levels (i.e., 1.34, 1.58, 1.82, 2.07, 2.31, 2.89, 3.64, and 4.13 mg N/L) were selected in this batch test set by controlling pH, and NO_2_^−^-N concentration, based on the documented formula^35^. The low FNA level used here is reported to effectively establish nitrite pathway in a nitrification-denitrification sludge^8^. Higher levels were chosen based on our hypothesis that a higher FNA concentration may be needed to suppress NOB in the absence of heterotrophic denitrification. Before FNA treatment, the original activities of AOB and NOB, which were determined as the specific ammonium oxidation and nitrate production rates on a VSS basis, were measured according to the method previously described^8^. After 24 h treatment, FNA in these batch reactors was removed through washing, and then the particle size distribution and activities of AOB and NOB were measured. The detailed procedure applied in batch test set I can be found in Supporting Information.

According to the results of the above batch tests, the suitable FNA concentration was determined to be either 1.82 or 3.64 mg N/L. To further determine the optimal treatment time and frequency, batch test set II was conducted at these two FNA levels. FNA treatment durations of 6, 12, 24, and 48 h were tested with each FNA level. Following treatment, the FNA in all reactors was removed by washing. The activities of AOB and NOB were measured after 0, 2, 4, and 6 d recovery, respectively (see Supporting Information for the detailed description).

Additional experiments (batch test set III) were also conducted to establish that FNA, rather than nitrite or pH, was responsible for the decreased NOB activity. Details of the experimental conditions can be found in Supporting Information.

### Mainstream reactor operation with FNA sludge treatment at 3.64 mg N/L (Phase II)

According to the batch test results, a treatment time of 24 h and a sludge treatment ratio of 25% (i.e. 25% of the sludge from the main reactor was treated everyday) were chosen. The mainstream reactor was operated as described for Phase I, with the exception that 2750 mL of sludge mixture (25% volume of mainstream reactor) was removed daily from the mainstream reactor at the end of an aerobic phase. The removed sludge was first thickened to 130 mL and then transferred into the FNA treatment unit. The pH in the unit was adjusted to 5.7 by addition of HCl, and a NaNO_2_ stock solution (60 g N/L) was added to result in a NO_2_^−^-N concentration of 750 mg N/L. This gives a calculated FNA concentration of 3.64 mg N/L (T = 22 °C). The pH was controlled at 5.7 ± 0.03 with a programmable logic controller using 0.5 M HCl solution and 0.5 M NaOH solution during the entire treatment process. After 24 h treatment, the FNA-treated sludge was returned to the mainstream reactor manually.

### Sludge treatment with FNA at 1.82 mg N/L (Phase III)

The mainstream reactor and the FNA treatment unit were operated as described in Phase II, except that the pH in the FNA treatment unit was adjusted to 6.0 (instead of 5.7), giving rise to an FNA concentration of 1.82 mg N/L (NO_2_^−^-N = 750 mg N/L; T = 22 °C).

### FNA treatment in combination with DO limitation (Phase IV)

The mainstream reactor and the FNA treatment unit were operated as in Phase III except that the DO concentration in the main reactor was lowered from 2.5–3.0 mg/L to 0.3–0.8 mg/L.

### Aeration length control for 50% ammonium conversion (Phase V)

To provide a suitable ammonium/nitrite ratio for subsequent anammox treatment, the aeration length in the mainstream reactor was switched off when approximately 50% of influent ammonium was oxidized. The control was implemented by adjusting the aeration time manually, with the amount of ammonium oxidized estimated based on the stoichiometric level of oxygen consumed. In large-scale applications, this should be better achieved by direct on-line ammonium measurement^36^. All other operation conditions were identical to those in Phase IV.

### Reduced sludge treatment frequency (Phase VI)

The mainstream reactor and the FNA treatment unit were operated as in Phase VI except that the amount of sludge mixture treated daily was reduced from 25% to 12.5%. The phase was designed to verify the effectiveness of the strategy with reduced treatment frequency.

### Analytical methods

The ammonium, nitrite and nitrate concentrations in the effluent were measured 2–4 times every week. Cycle studies were conducted every week by measuring the ammonium, nitrite and nitrate concentrations throughout a cycle. TSS and VSS concentrations were also determined weekly. At the end of each phase (except for Phase II), fluorescence *in-situ* hybridization (FISH) was performed to quantify the populations of NOB and AOB.

The analyses of COD, TSS, and VSS were performed in accordance with standard methods^37^. The concentrations of ammonium, nitrite, and nitrate were measured using a Lachat QuikChem8000 Flow Injection Analyzer (Lachat Instrument, Milwaukee, Wisconsin). Particle size distribution was analyzed using Mastersizer 2000 series (Malvern Instruments, Worcestershire, UK) on the basis of volumetric distribution according to the method previously described^38^. FISH was employed to quantify AOB and NOB abundances in sludge. The following oligonucleotide probes were used: NSO1225 (specific for Betaproteobacterial AOB and labelled with Cy5), Ntspa662 and Ntspa712 (specific for the *Nitrospira* sp. and labelled with Cy3), NIT3 (specific for *Nitrobacter* sp. and labelled with Cy3), and EUB-mix (specific for total bacteria and labelled with FITC)^28^. To get the changes of NOB composition, the oligonucleotide probes NIT3, Ntspa662 and Ntspa712, and EUB-mix, which were respectively labelled with Cy5, Cy3, and FITC, were also used in this study. FISH was performed according to the method previously described^39^,^40^. For quantitative FISH analysis, at least 20 random microscopic fields from several layers were captured for each probing event using a Zeiss LSM 510 Meta confocal laser scanning microscope (Carl Zeiss, Jena, Germany) and image analysis was performed using the program daime. Each probing event was expressed as a percentage of the total area detected with the EUBmix probes.

## Additional Information

**How to cite this article**: Wang, D. *et al.* Achieving Stable Nitritation for Mainstream Deammonification by Combining Free Nitrous Acid-Based Sludge Treatment and Oxygen Limitation. *Sci. Rep.* **6**, 25547; doi: 10.1038/srep25547 (2016).

## Supplementary Material

Supplementary Information

## Figures and Tables

**Table 1:** Conditions and Activity of AOB and NOB in Batch Test I after 24 h Treatment at Different Levels of FNA^a^.

FNA (mg/L)	NO2-N (mg/L)	pH	°C	Treatment time (h)	AOB activity (% of the original)	NOB activity (% of the original)
1.34	550	6.0	22	24	82.0	75.3
1.58	650	6.0	22	24	70.4	68.0
1.82	750	6.0	22	24	73.1	43.5
2.07	850	6.0	22	24	77.9	52.0
2.31	950	6.0	22	24	69.3	44.9
2.89	750	5.8	22	24	64.7	39.7
3.64	750	5.7	22	24	58.3	6.7
4.13	850	5.7	22	24	51.0	4.9

^a^The original activities of AOB and NOB were 67.2 and 49.4 mg N/g VSS·h.

**Table 2:** Performance of the Mainstream Reactor in Steady-state Operation of Different Phases^a^.

		Phase I	Phase III	Phase IV	Phase V	Phase VI
Effluent NH_4_^+^-N	Concentration (mg/L)	0.34 ± 0.03	0.79 ± 0.28	0.88 ± 0.17	26.6 ± 0.7	27.1 ± 1.4
	Ratio (%)^b^	0.6 ± 0.1	1.3 ± 0.5	1.6 ± 0.3	48.6 ± 1.5	47.5 ± 2.1
Effluent NO_2_^−^-N	Concentration (mg/L)	1.36 ± 0.42	34.9 ± 0.5	43.6 ± 1.1	26.0 ± 0.5	26.9 ± 0.6
	Ratio (%)^c^	2.4 ± 0.8	58.5 ± 0.3	78.9 ± 1.1	47.6 ± 0.5	47.1 ± 1.3
Effluent NO_3_^−^-N	Concentration (mg/L)	55.6 ± 1.7	23.9 ± 0.9	10.8 ± 0.6	2.07 ± 0.55	3.13 ± 0.45
	Ratio (%)^d^	97.0 ± 0.7	40.2 ± 0.7	19.5 ± 1.1	3.8 ± 1.0	5.4 ± 0.8
Effluent soluble COD (mg/L)		7 ± 1	5 ± 1	6 ± 1	6 ± 1	5 ± 1
TSS (mg/L)		392 ± 28	192 ± 13	171 ± 5	114 ± 10	117 ± 4
VSS (mg/L)		365 ± 30	172 ± 12	157 ± 6	103 ± 9	106 ± 3

^a^Results are the averages and standard deviations from triplicate measurements.

^b^Effluent NH_4_^+^-N ratio = Effluent NH_4_^+^-N/Effluent total nitrogen × 100%.

^c^Effluent NO_2_^−^-N ratio = Effluent NO_2_^−^-N/Effluent total nitrogen × 100%.

^d^Effluent NO_3_^−^-N ratio = Effluent NO_3_^−^-N/Effluent total nitrogen × 100%.

**Table 3:** AOB and NOB Abundance and Activity in the Mainstream Reactor at Steady-State in Different Phases^a^.

	Phase I	Phase III	Phase IV	Phase V	Phase VI
AOB population (%)	58.5 ± 5.4	71.7 ± 7.2	76.1 ± 4.9	81.3 ± 6.4	80.5 ± 6.1
AOB activity (mg N/g VSS·h)	67.2 ± 4.6	59.5 ± 3.8	73.9 ± 1.5	68.2 ± 3.4	67.3 ± 3.9
NOB population (%)	23.0 ± 4.3	5.3 ± 1.9	2.1 ± 1.0	0.7 ± 0.1	0.9 ± 0.1
*Nitrospira* abundance (%)	15.7 ± 3.9	0.4 ± 0.1	0.3 ± 0.1	ND^b^	ND^b^
*Nitrobacter* abundance (%)	7.3 ± 2.6	4.9 ± 1.2	1.8 ± 0.8	0.7 ± 0.1	0.9 ± 0.1
NOB activity (mg N/g VSS·h)	49.4 ± 3.1	24.9 ± 1.9	9.8 ± 2.6	7.5 ± 2.1	7.8 ± 1.7

^a^Results are the averages and standard deviations from triplicate measurements.

^b^ND = non-detectable.

**Table 4:** Variations of Total COD, Soluble COD, Ammonium, Nitrite, Nitrate, and Activities of AOB and NOB in FNA Treatment Unit^a^.

Treatment time	Total COD (mg/L)	Soluble COD (mg/L)	Ammonium (mg/L)	Nitrite (mg/L)	Nitrate (mg/L)	AOB activity (mg N/g VSS·h)	NOB activity (mg N/g VSS·h)
0	2560 ± 115	6 ± 2	0.12 ± 0.02	767 ± 28	1.18 ± 0.06	59.5 ± 3.8	24.9 ± 1.9
24 h	2548 ± 132	327 ± 21	0.19 ± 0.03	761 ± 23	0.92 ± 0.04	47.1 ± 2.6	11.7 ± 0.8

^a^Results are the averages and standard deviations from triplicate measurements during steady-state in Phase III. The pH in the FNA treatment unit was 6.0 during the entire treatment period.
