# Supplementary material for: Achieving Stable Nitritation for Mainstream Deammonification by Combining Free Nitrous Acid-Based Sludge Treatment and Oxygen Limitation
Source: Sci Rep. 2016 May 6;6:25547. doi: 10.1038/srep25547 (PMC4858757; doi:10.1038/srep25547)
Supplement: Supplementary Information [file srep25547-s1.doc]

Supporting Information

**Achieving Stable Nitritation for Mainstream Deammonification by Combining Free Nitrous Acid-Based Sludge Treatment and Oxygen Limitation**

Dongbo Wang, Qilin Wang, Andrew Laloo, Yifeng Xu, Philip L. Bond, Zhiguo Yuan*

*Advanced Water Management Centre (AWMC), The University of Queensland, QLD 4072, Australia*

Corresponding author

Tel: +61-7-33654374

Fax: +61-7-33654726

E-mail: [zhiguo@awmc.uq.edu.au](mailto:zhiguo@awmc.uq.edu.au)

The Number of Figures: 5

The Number of Tables: 2

**MATERIALS AND METHODS**

**Batch Test Set I:** A total of 900 mL sludge was taken from the mainstream reactor at the end of an aerobic period during its steady-state operation in Phase I. The sample was divided equally into 9 batch reactors each with a working volume of 100 mL. All reactors were mixed with stirrers at a speed of 120 rpm in a 22 ± 1 ℃ temperature-controlled room. In one reactor, the activities of AOB and NOB were immediately measured before FNA treatment according to the method previously described ^1^. Briefly, the mixed liquor was first washed using the effluent (nitrite free) from the SBR to remove nitrite. Then, the stock solutions of ammonium and nitrite were added into these batch reactors, reaching an initial ammonium and nitrite concentration of 25 mg NH_4_^+^-N/L and 25 mg NO_2_^-^-N/L, respectively. The DO was not limiting (DO > 5 mg/L) while the pH was controlled between 7.2 and 7.5 manually during the whole experimental period. The volumetric ammonium oxidation and nitrate production rates were determined through linear regression of the corresponding profiles. The specific ammonium oxidation and nitrate production rates were calculated by dividing the corresponding volumetric rates by the VSS concentration. After that, different volumes of NaNO_2_ stock solution (60 g N/L) were respectively added into the other 8 reactors to achieve the designated nitrite concentrations (i.e., 550, 650, 750, 850, 950, 750, 750, and 850 mg N/L). The pH in these 8 reactors was respectively controlled at 6.0, 6.0, 6.0, 6.0, 6.0, 5.8, 5.7, and 5.7 via programmable logic controllers using 0.5 M HCl solution and 0.5 M NaOH solution during the whole treatment process. The temperature, pH and NO_2_^-^-N concentration applied here led to FNA concentrations of 1.34, 1.58, 1.82, 2.07, 2.31, 2.89, 3.64, and 4.13 mg N/L in these reactors, respectively, based on the formula documented in the literature ^2^. After 24 h treatment, FNA in these reactors was removed through washing, and then the particle size distribution and activities of AOB and NOB were measured.

**Batch Test Set II:** A total of 800 mL of sludge was withdrawn from the mainstream reactor at the end of the aerobic period and then distributed evenly into 8 identical batch reactors (100 mL). These reactors were divided equally into two groups with four reactors each. The FNA concentration of group I was adjusted to 1.82 mg N/L (nitrite = 750 mg/L; pH = 6.0) while that for group II was 3.64 mg N/L (nitrite = 750 mg/L; pH = 5.7). The FNA treatment duration in each group was 6, 12, 24, 48 h, respectively. All other operations were the same as those described above (Set I). After FNA treatment, FNA in all reactors was removed through washing, then the activities of AOB and NOB were measured at 0, 2, 4 and 6 d after recovery started. During the recovery period, air was pumped into all reactors to ensure that DO was not limiting (DO > 5 mg/L) while pH was controlled at 7.5 ± 0.1 by dosing 0.5 M HCl and 0.5 M NaOH. The initial NH_4_^+^-N and NO_2_^-^-N concentrations were about 35 mg/L by adding stock solutions of ammonium and nitrite. To avoid the absence of NH_4_^+^-N (due to consumption) and the accumulation of NO_2_^-^-N, the mixture in all reactors was centrifuged and the supernatant was replaced with 35 mg/L solution of ammonium and nitrite twice per day.

**Batch Test Set III:** 3 identical reactors, which were respectively defined as pH6-reactor, nitrite-reactor, and FNA-reactor, was performed to provide such support. The nitrite concentration in the nitrite-reactor and FNA-reactor was controlled at 750 mg/L while no nitrite was added into the pH 6-reactor. The pH in the FNA-reactor and pH 6-reactor was maintained at 6. To minimize the effect of FNA, the pH in the nitrite-reactor was maintained at 8.5, which resulted in the FNA level of only 5.8 × 10^-3^ mg/L). All other operations were the same as described in the batch test set I.

**TABLES**

| **Table 1. Results of Batch Test II to Determine the Effect of Different Treatment and Recovery Time on the Activity of AOB and NOB at 1.82 and 3.64 mg N/L of FNA Concentrations ^a^** | | | | | | | |
| --- | --- | --- | --- | --- | --- | --- | --- |
| Treatment time (h) | Recovery time (d) |  | 1.82 mg N/L FNA | |  | 3.64 mg N/L FNA | |
|  |  |  | AOB activity  (% of the original) | NOB activity  (% of the original) |  | AOB activity  (% of the original) | NOB activity  (% of the original) |
|  |  |  |  |  |  |  |  |
| 6 | 0 |  | 83.0 | 77.3 |  | 64.6 | 44.3 |
| 6 | 2 |  | 87.6 | 84.0 |  | 61.9 | 45.5 |
| 6 | 4 |  | 91.2 | 87.4 |  | 73.2 | 50.1 |
| 6 | 6 |  | 93.0 | 89.9 |  | 79.5 | 74.5 |
|  |  |  |  |  |  |  |  |
| 12 | 0 |  | 70.4 | 51.8 |  | 55.8 | 23.5 |
| 12 | 2 |  | 76.3 | 63.4 |  | 57.9 | 37.7 |
| 12 | 4 |  | 79.3 | 70.4 |  | 62.8 | 43.1 |
| 12 | 6 |  | 83.2 | 84.8 |  | 73.7 | 69.2 |
|  |  |  |  |  |  |  |  |
| 24 | 0 |  | 68.0 | 40.3 |  | 53.4 | 5.9 |
| 24 | 2 |  | 65.2 | 47.2 |  | 61.5 | 12.9 |
| 24 | 4 |  | 72.0 | 50.8 |  | 61.9 | 16.0 |
| 24 | 6 |  | 75.1 | 72.5 |  | 69.5 | 28.9 |
|  |  |  |  |  |  |  |  |
| 48 | 0 |  | 66.5 | 34.8 |  | 11.3 | 3.0 |
| 48 | 2 |  | 68.9 | 41.1 |  | 12.8 | 3.4 |
| 48 | 4 |  | 69.6 | 50.4 |  | 22.6 | 3.6 |
| 48 | 6 |  | 76.3 | 64.9 |  | 36.2 | 5.7 |
| ^a^ The original activities of AOB and NOB were 67.2 and 49.4 mg N/g VSS•h. | | | | | | | |

| **Table S2. AOB and NOB Activities after 24 h Treatment with pH 6, 750 mg/L Nitrite, and 1.82 mg/L FNA ^a^** | | | |
| --- | --- | --- | --- |
|  | pH 6-reactor | Nitrite-reactor | FNA-reactor |
| AOB activity (mg/g VSS·h) | 64.8 ± 3.5 | 66.5 ± 3.8 | 47.2 ± 2.4 |
| NOB activity (mg/g VSS·h) | 47.8 ± 2.9 | 48.9 ± 2.1 | 20.3 ± 1.3 |
| ^a^ Results are the averages and their standard deviations of triplicate measurements. The original activities of AOB and NOB were 67.2 ± 4.6 and 49.4 ± 3.1 mg N/g VSS•h. | | | |

| **Table S3. Case Analyses of the FNA-Based Strategy Supporting Maximal Energy Recovery and Desirable Nutrient Removal in a WWTP (10000 m^3^/d).** | |
| --- | --- |
| **General Parameters** |  |
| Size of the WWTP (Population equivalent - PE) | 200,000 |
| Decay coefficient of the heterotrophic biomass (d^-1^) | 0.2 ^a^ |
| Decay coefficient of the nitrifying biomass (d^-1^) | 0.1 ^a^ |
| Yield coefficient of the heterotrophic biomass (g COD/g COD) | 0.625 ^a^ |
| Yield coefficient of the nitrifying biomass (g COD/g N) | 0.24 ^a^ |
| Fraction of inert COD generated in biomass decay (g COD/g COD) | 0.2 ^a^ |
| **WWTP with conventional operation** |  |
| **VSS production (t VSS/y)** | 0.91 × 10^3^ |
| Destruction of waste activated sludge in anaerobic digester (VS basis) | 35% ^a^ |
| **Methane production (kg CH_4_/y)** | 1.7 × 10^5^ |
| **WWTP with FNA-supported mainstream deammonification operation** |  |
| TSS concentration in the A-stage (mg/L) | 2,000 |
| VSS concentration in the A-stage (mg/L) | 1,800 |
| SRT in the A-stage (d) | 0.5 ^b^ |
| Nitrogen removal ratio in A-stage (%) | 30 |
| Organic carbon removal ratio in A-stage (%) | 80 ^c^ |
| TSS in the partial nitritation reactor (mg/L) | 2,000 |
| VSS in the partial nitritation reactor (mg/L) | 1,700 |
| SRT in the mainstream nitritation reactor (d) | 8 ^d^ |
| Sludge ratio wasted for FNA treatment (%) | 12.5 ^d^ |
| Nitrite accumulation ratio in the mainstream nitritation reactor (%) | 48 ^d^ |
| Solids content in thickened sludge (%) | 3 |
| Solids content in dewatered sludge (%) | 15 |
| Destruction of A-stage sludge in anaerobic digester (VS basis, %) | 70 ^c^ |
| **VSS production at A-stage (t VSS/y)** | 1.02 × 10^3^ |
| Destruction of nitritation sludge in anaerobic digester (VS basis, %) | 35 ^a^ |
| **VSS production at B-stage (t VSS/y)** | 0.22 × 10^3^ |
| Nitrite accumulation ratio in the nitrite production reactor (%) | 90 ^e^ |
| Conversion ratio of ammonium (%) | 50 |
| **Methane production (kg CH_4_/y)** | 8 × 10^5^ |
| Achievable nitrite concentration in the FNA treatment unit (mg N/L) | 750 |
| **The pH required to be controlled** | 6.0 ^d^ |

^a^ See reference: Metcalf and Eddy. Wastewater Engineering: Treatment and Reuse. McGraw-Hill Inc.: 2003.

^b^ See reference: Kampschreur, M. J.; van der Star, W. R. L.; Wielders, H. A.; Mulder, J. W.; Jetten, M. S. M.; van Loosdrecht, M. C. M. Dynamics of nitric oxide and nitrous oxide emission during full-scale reject water treatment. Water Res. 2008, 42, 812-826.

^c^ See reference: Ge, H.; Batstone, D. J.; Keller, J. Operating aerobic wastewater treatment at very short sludge ages enables treatment and energy recovery through anaerobic sludge digestion. Water Res. 2013, 47, 6546-6557.

^d^ The data were obtained in this work.

^c^ See reference: Law, Y.; Ye, L.; Wang, Q.; Hu, S.; Pijuan, M.; Yuan, Z. Producing free nitrous acid - A green and renewable biocidal agent - From anaerobic digester liquor. Chem. Eng. J. 2015, 259, 62-69.

^d^ This value is calculated by the formula FNA = S_NO2--N_/(K_a_ × 10 ^pH^) with K_a_ value determined by the formula K_a_ = e (-2300/(T+273)) for a given temperature T (°C). Where, the required FNA concentration is 1.82 mg/L, temperature is 22 °C, and nitrite concentration is 750 mg/L.

**FIGURES**

**
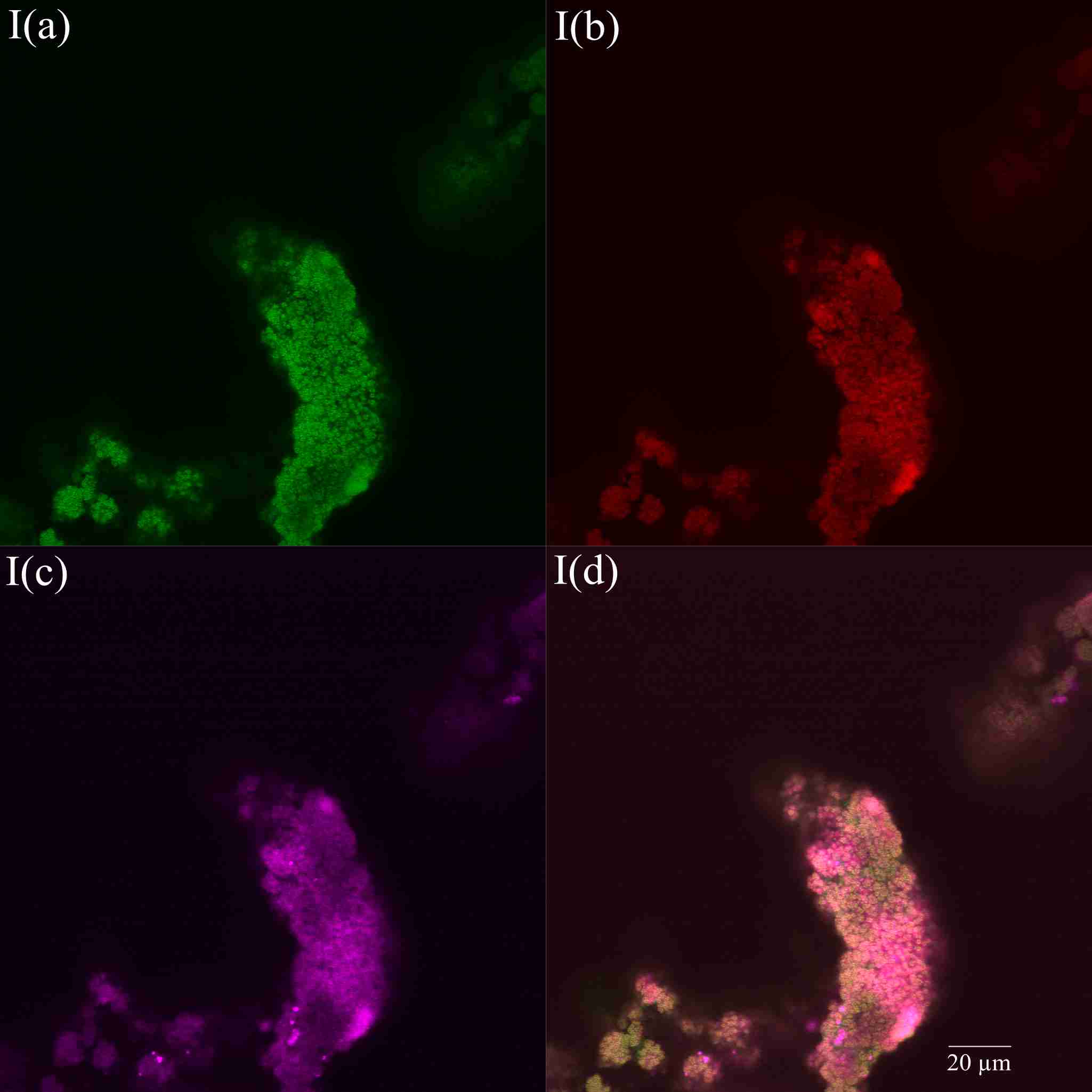

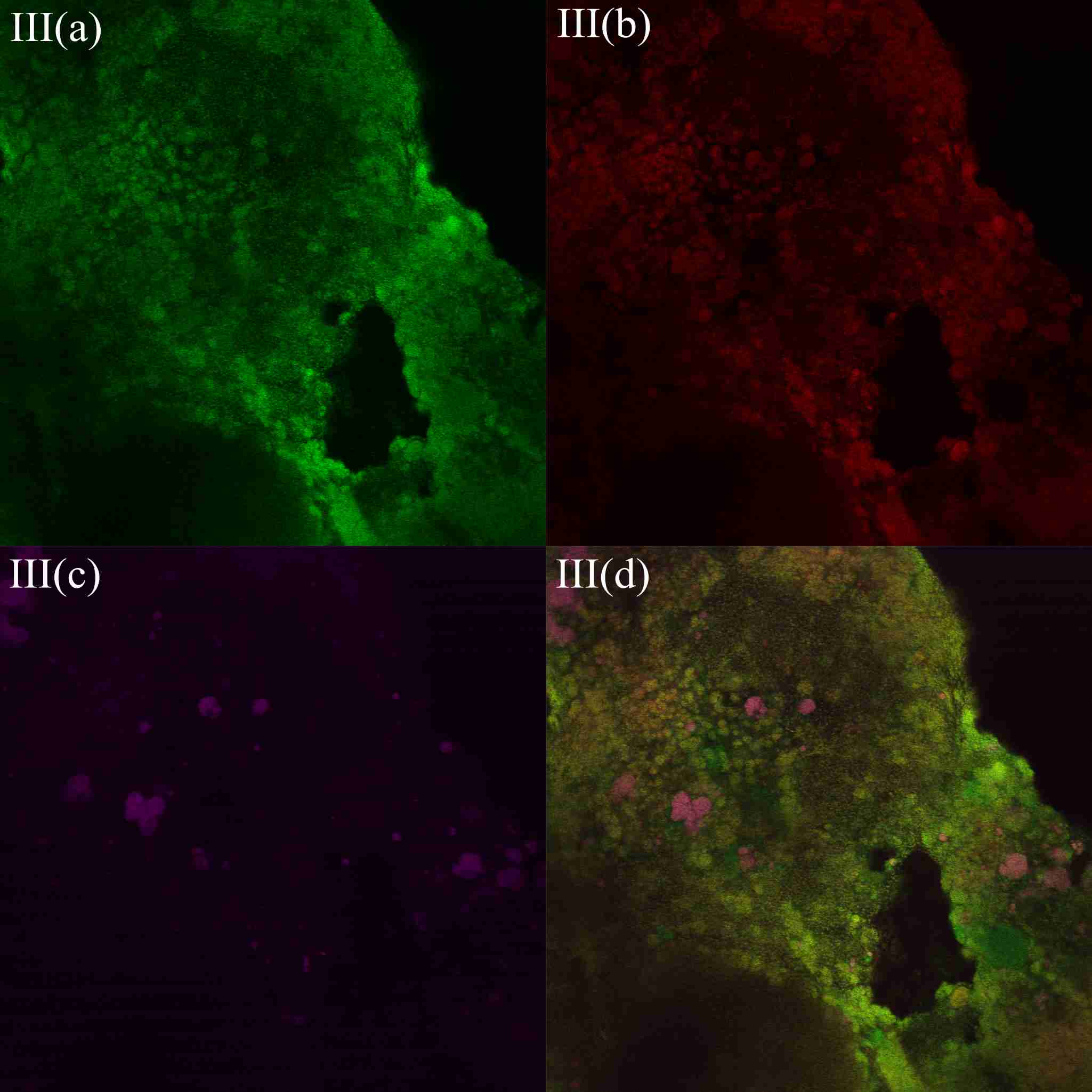
**

**
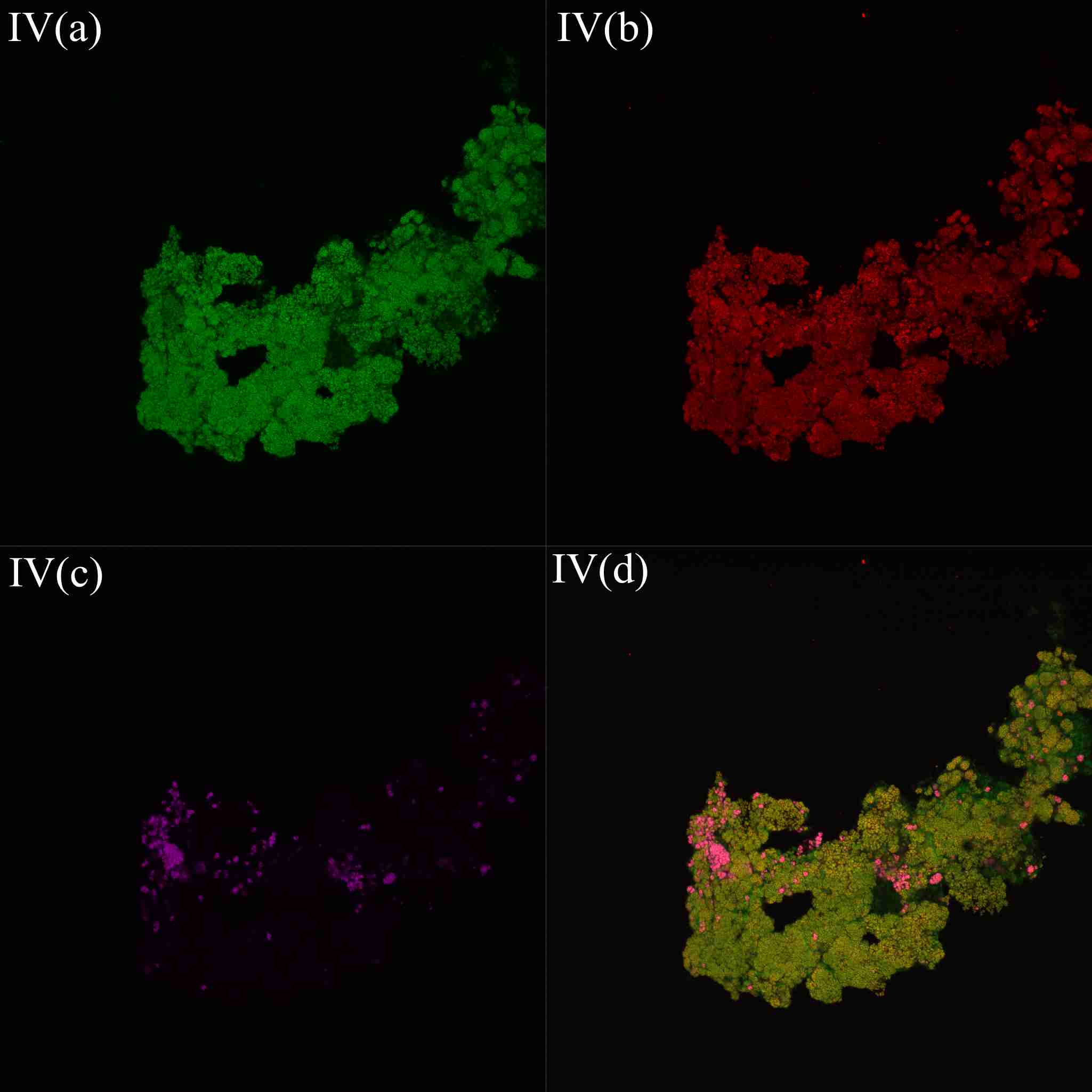

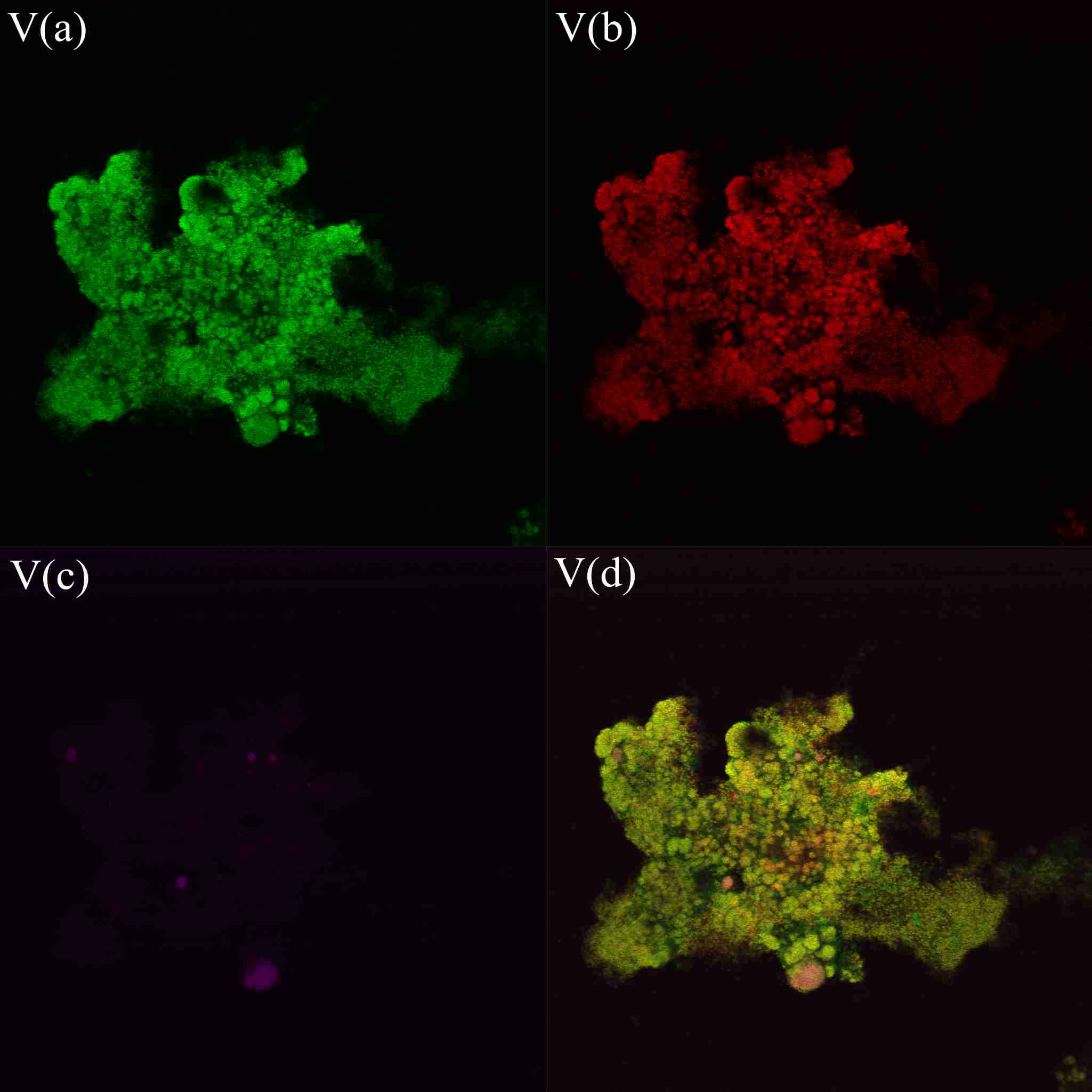
**

**
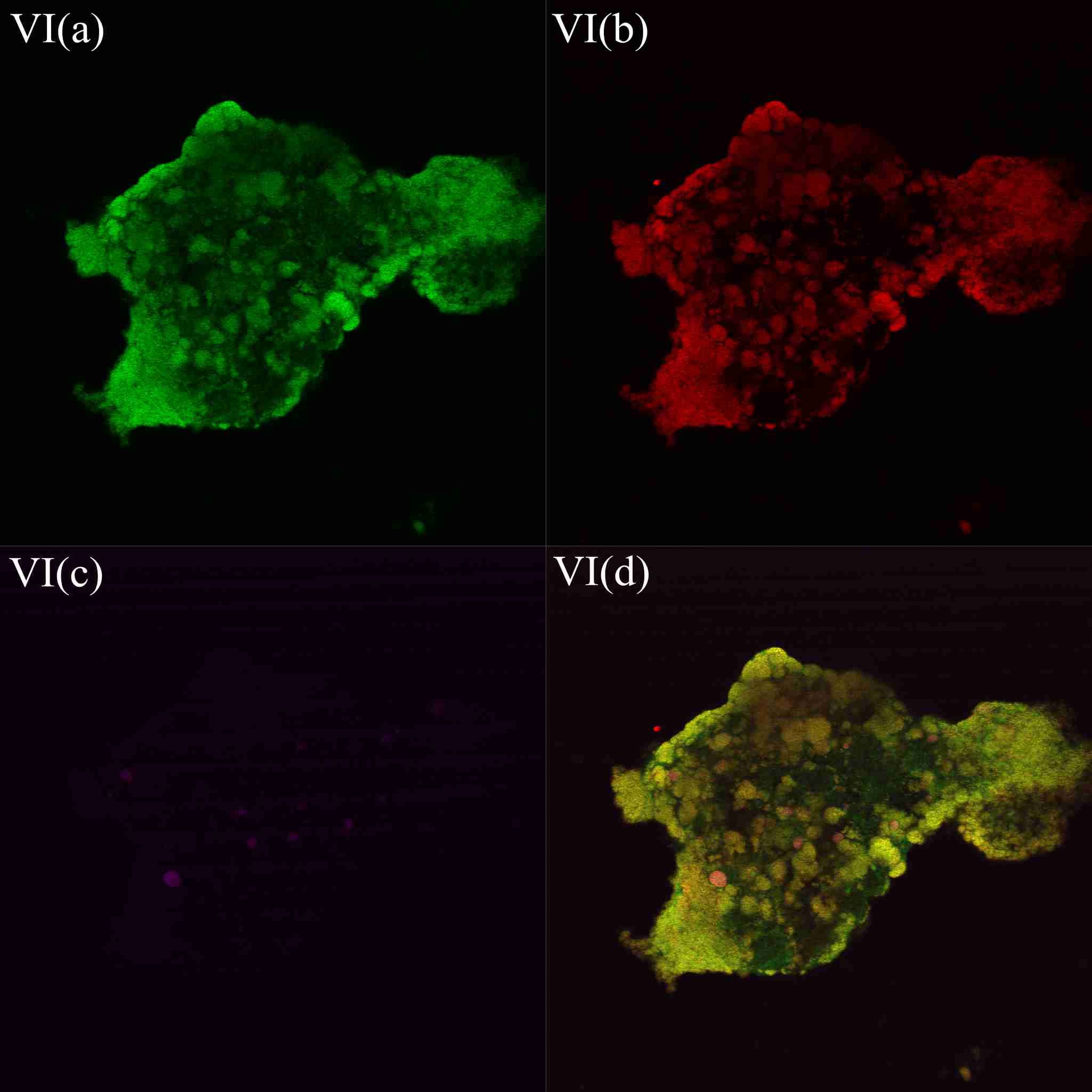
**

**Figure S1. FISH images of microbial community from the mainstream reactor under different operational conditions (at steady-state in each phase) hybridized with EUBmix (green, a), NSO1225 (red, b), and NIT3 and Ntspa662 and Ntspa712 (purple, c) probes, respectively. An overlay of all these probes is shown in d. I: Phase I; III: Phase III; IV: Phase IV; V: Phase V; VI: Phase VI.**


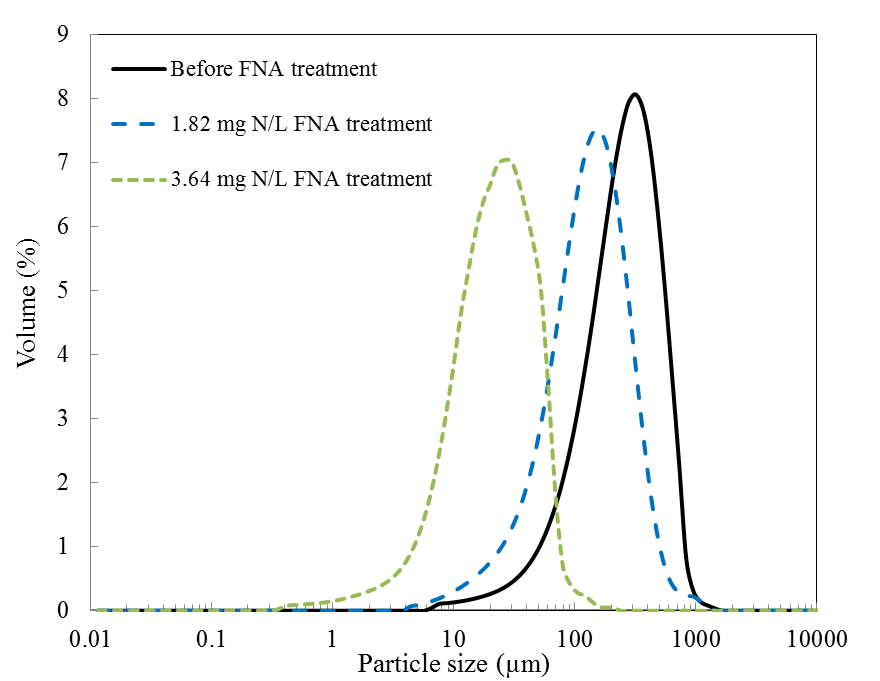


**Figure S2. The distribution of particle size in the original sludge, sludge treated with FNA at 1.82 mg N/L for 24 h and sludge treated with FNA at 3.64 mg N/L for 24 h.**

**Figure S3. The long-term variations of TSS and VSS in the mainstream reactor (A) and in the effluent (B) in different phases.**

**Figure S4. The cycle variations of ammonium, nitrite, and nitrate in each phase during steady-state operation except for Phase II. A: Phase I; B: Phase II (on day 61); C: Phase III; D: Phase IV; E: Phase V; D: Phase VI.**

**
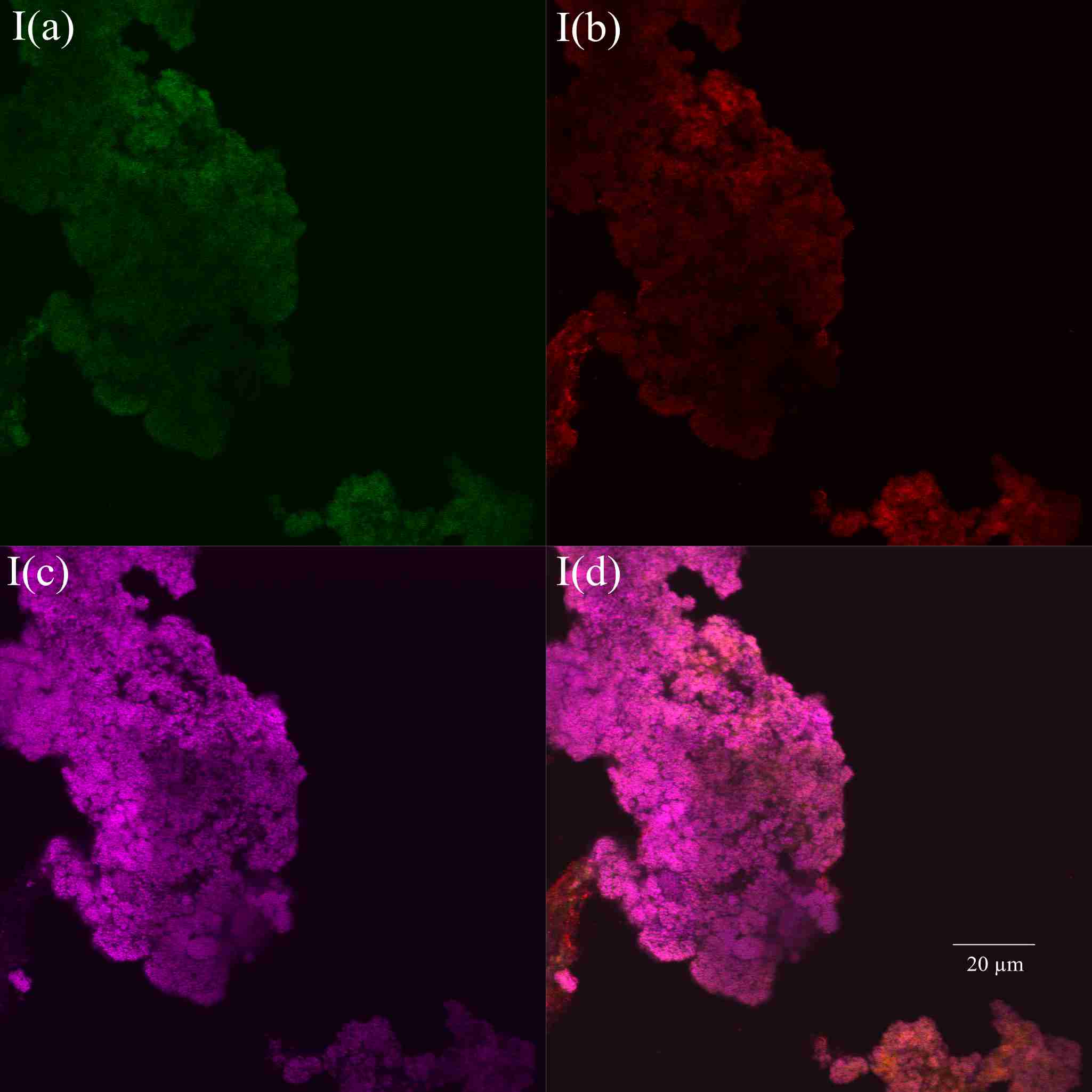

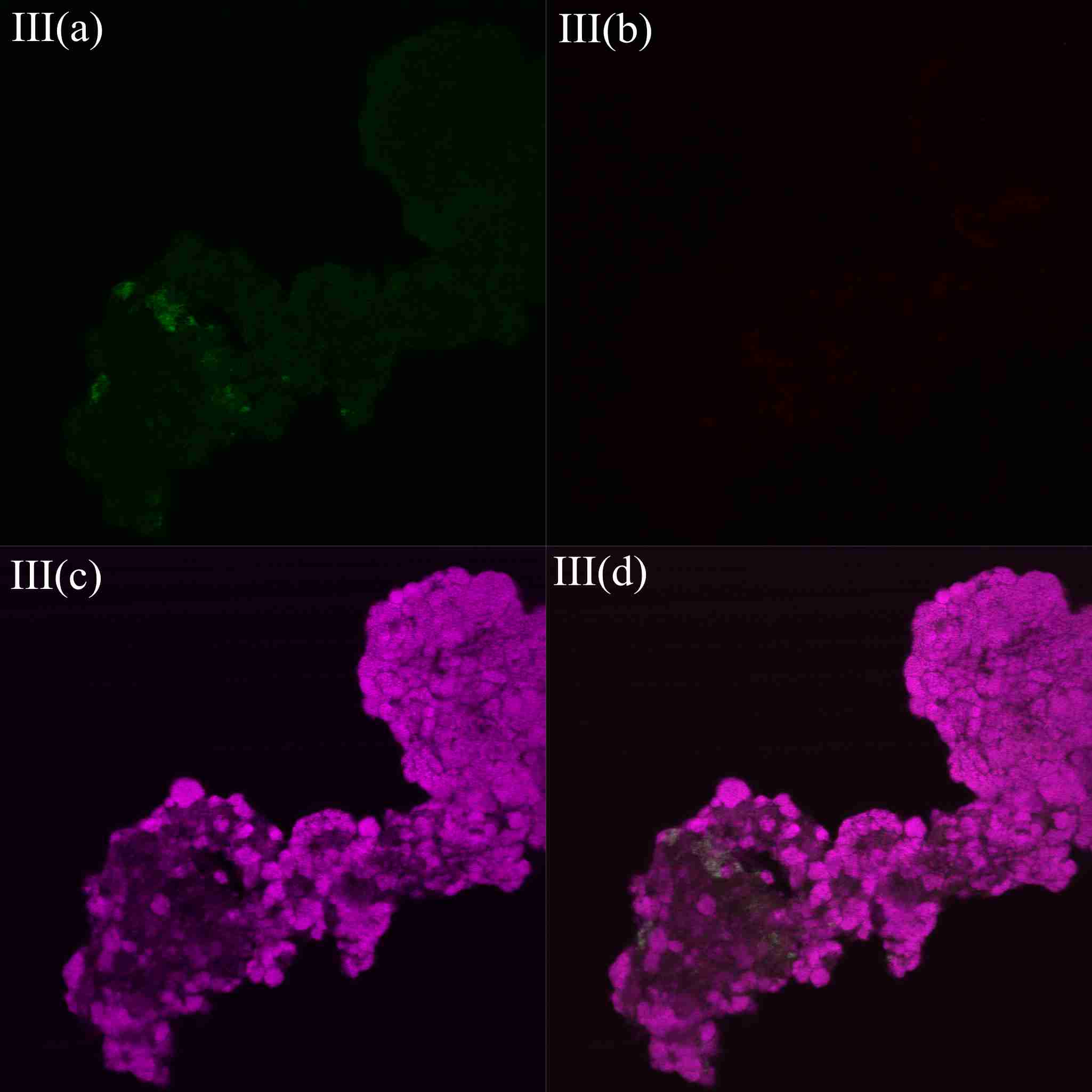
**

**
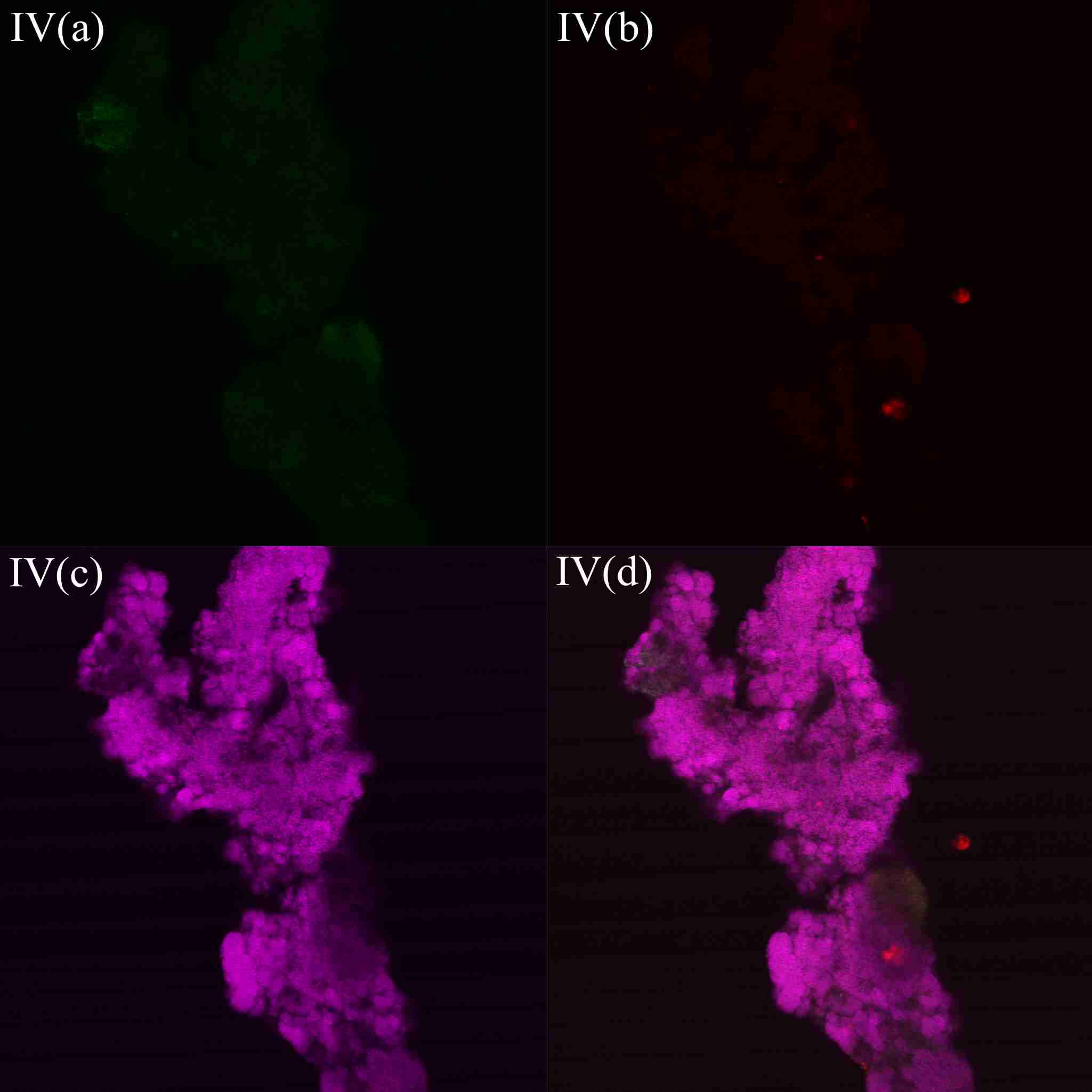

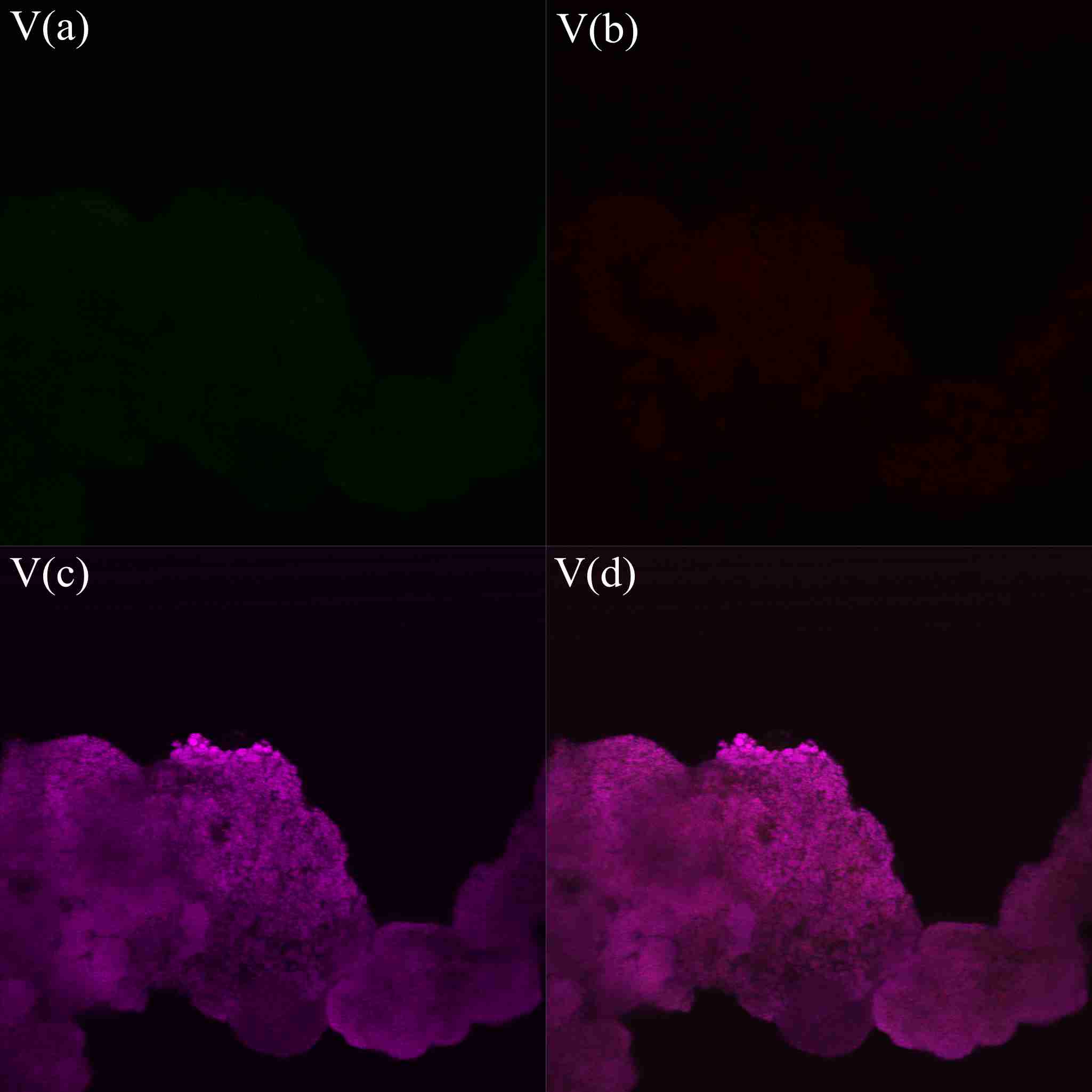
**

**
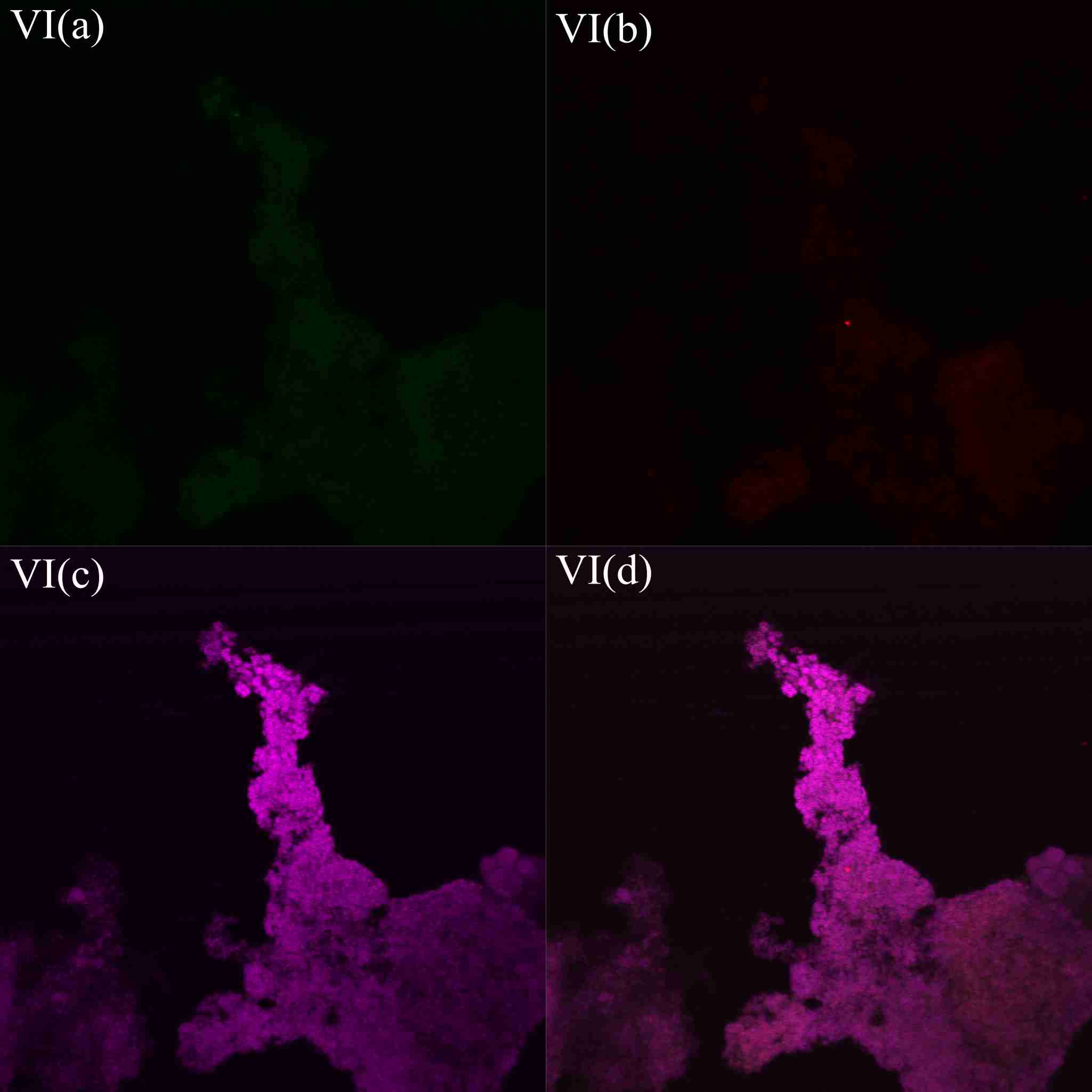
**

**Figure S5. FISH images of microbial community from the mainstream reactor under different operational conditions (at steady-state operation) hybridizing with NIT3 (green, a), Ntspa662 and Ntspa712 (red, b), and EUBmix (purple, c) probes, respectively. An overlay of all these probes is shown in d. I: Phase I; III: Phase III; IV: Phase IV; V: Phase V; VI: Phase VI.**

**REFERENCES**

1. Wang, Q., Ye, L., Jiang, G., Hu, S. & Yuan, Z. Side-stream sludge treatment using free nitrous acid selectively eliminates nitrite oxidizing bacteria and achieves the nitrite pathway. *Water Res* **55**, 245-255 (2014).
2. Anthonisen, A. C., Loehr, R. C., Prakasam, T. B. S. & Shinath, E. G. Inhibition of nitrification by ammonia and nitrous acid. *J Water Pollut Control Fed* **48**, 835-852 (1976).
